# Effects of norepinephrine on tissue perfusion in a sheep model of intra-abdominal hypertension

**DOI:** 10.1186/s40635-015-0046-1

**Published:** 2015-03-31

**Authors:** Gonzalo Ferrara, Vanina S Kanoore Edul, Juan F Caminos Eguillor, Enrique Martins, Carlos Canullán, Héctor S Canales, Can Ince, Elisa Estenssoro, Arnaldo Dubin

**Affiliations:** Academic Medical Center, Department of Translational Physiology, University of Amsterdam, Meibergdreef 9, 1105 Amsterdam, AZ The Netherlands; Cátedra de Farmacología Aplicada, Facultad de Ciencias Médicas, Universidad Nacional de La Plata, 60 y 120, 1900 La Plata, Argentina

**Keywords:** Intra-abdominal hypertension, Perfusion pressure, Microcirculation, Renal blood flow, Urinary output

## Abstract

**Background:**

The aim of the study was to describe the effects of intra-abdominal hypertension (IAH) on regional and microcirculatory intestinal blood flow, renal blood flow, and urine output, as well as their response to increases in blood pressure induced by norepinephrine.

**Methods:**

This was a pilot, controlled study, performed in an animal research laboratory. Twenty-four anesthetized and mechanically ventilated sheep were studied. We measured systemic hemodynamics, superior mesenteric and renal blood flow, villi microcirculation, intramucosal-arterial PCO_2_, urine output, and intra-abdominal pressure. IAH (20 mm Hg) was generated by intraperitoneal instillation of warmed saline. After 1 h of IAH, sheep were randomized to IAH control (*n* = 8) or IAH norepinephrine (*n* = 8) groups, for 1 h. In this last group, mean arterial pressure was increased about 20 mm Hg with norepinephrine. A sham group (*n* = 8) was also studied. Fluids were administered to prevent decreases in cardiac output. Differences between groups were analyzed with two-way repeated measures of analysis of variance (ANOVA).

**Results:**

After 2 h of IAH, abdominal perfusion pressure decreased in IAH control group compared to IAH norepinephrine and sham groups (49 ± 11, 73 ± 11, and 86 ± 15 mm Hg, *P* < 0.0001). There were no differences in superior mesenteric artery blood flow, intramucosal-arterial PCO_2_, and villi microcirculation among groups. Renal blood flow (49 ± 30, 32 ± 24, and 102 ± 45 mL.min^−1^.kg^−1^, *P* < 0.0001) and urinary output (0.3 ± 0.1, 0.2 ± 0.2, and 1.0 ± 0.6 mL.h^−1^.kg^−1^, *P* < 0.0001) were decreased in IAH control and IAH norepinephrine groups, compared to the sham group.

**Conclusions:**

In this experimental model of IAH, the gut and the kidney had contrasting responses: While intestinal blood flow and villi microcirculation remained unchanged, renal perfusion and urine output were severely compromised.

**Electronic supplementary material:**

The online version of this article (doi:10.1186/s40635-015-0046-1) contains supplementary material, which is available to authorized users.

## Background

The occurrence of elevated intra-abdominal pressure (IAP) has been increasingly recognized in critically ill patients as a complication of several abdominal and extra-abdominal conditions and also as an independent predictor of mortality [1]. Sustained or repeated values of IAP ≥12 mm Hg define intra-abdominal hypertension (IAH). IAH may affect organ function and lead to the abdominal compartment syndrome, which is characterized by a sustained IAP >20 mm Hg plus the development of new organ dysfunctions/failures [2].

The involvement of intra-abdominal organs during IAH is heterogeneous. Clinical and experimental studies showed that the kidney is particularly susceptible**,** even to small increases in IAP [3], evidenced by decreased glomerular filtration and oliguria. In contrast, the gut compromise in patients with IAH seems to be variable: reports go from overt ischemia with bowel infarction [4], to subtle hypoperfusion generating mucosal barrier dysfunction [5] or intramucosal acidosis [6]. Experimental research also showed dissimilar gut alterations. Some studies found severe reductions in gut perfusion induced by an IAP of 15 mm Hg maintained for 2 h [7], while others showed preservation of intestinal blood flow after 3 h of 20 mm Hg [8]. These contradictory findings might be related to different models of IAH but also to the particular behavior of cardiac output in each animal model. Therefore, the decrease in regional and tissue intestinal blood flow might not be completely dissociated from the alterations in systemic hemodynamics.

The abdominal perfusion pressure (APP) is the difference between mean arterial pressure (MAP) and IAP. The APP is a surrogate of the driving pressure for the perfusion of intra-abdominal organs; and its decrease might thus be the major pathophysiologic mechanism underlying regional hypoperfusion and organ failures. The APP was suggested as a more accurate predictor of visceral perfusion and a better goal of resuscitation than isolated values of IAP or MAP [9]; yet, there is no clinical evidence supporting an APP above 60 mm Hg as a therapeutic goal. Accordingly, the World Society for Abdominal Compartment Syndrome makes no recommendation regarding the use of APP in the resuscitation of critically ill patients with IAH [10].

Few experimental studies have evaluated the benefits of increasing MAP on tissue perfusion. Hence, our goal was to describe the effects of IAH on regional and microvascular intestinal perfusion, and on renal blood flow and urinary output, in an adequately resuscitated sheep model. Another aim was to assess the effect of an increase in APP, mediated by norepinephrine.

Our hypothesis was that the IAH produces a heterogeneous compromise in the circulation of intra-abdominal organs and that norepinephrine restores such disorders.

## Methods

This study was approved by the local Animal Research Committee [0800-009634/11-000]. Care of animals was in accordance with National Institutes of Health (United States).

### Surgical preparation

Twenty-four sheep (mean weight, 21 kg (SD, 4 kg)) were anesthetized with 30 mg.kg^−1^ of sodium pentobarbital, intubated, and mechanically ventilated with a Servo Ventilator 900C (Siemens-Elema AB, Solna, Sweden) with a tidal volume of 15 mL.kg^−1^, an FiO_2_ of 0.21, and a positive end-expiratory pressure of 6 cm H_2_O. The initial respiratory rate was set to keep the arterial PCO_2_ between 35 and 40 mm Hg. This respiratory setting was maintained during the rest of the experiment. Neuromuscular blockade was performed with pancuronium bromide (0.06 mg.kg^−1^). Additional pentobarbital boluses (1 mg.kg^−1^) were administered hourly and when clinical signs of inadequate depth of anesthesia were evident. Analgesia was provided by fentanyl as a bolus of 2 μg.kg^−1^, followed by 1 μg.h^−1^.kg^−1^. These drugs were intravenously administered.

A 7.5 French Swan-Ganz Standard Thermodilution Pulmonary Artery Catheter (Edwards Life Sciences, Irvine, CA, USA) was inserted through an introducer in the right external jugular vein; its side port was used to administer fluids and drugs. A catheter was placed in the descending aorta via the left femoral artery to measure blood pressure and obtain blood samples.

A midline laparotomy was performed, followed by a gastrostomy to drain gastric contents, and a splenectomy to avoid spleen contraction.

Perivascular ultrasonic flow probes were placed around the superior mesenteric artery and the left renal artery, to measure intestinal and renal arterial blood flows.

A catheter was situated in the superior mesenteric vein through a small vein proximal to the gut to draw blood samples. A tonometer was inserted through a small ileotomy to measure intramucosal PCO_2_.

IAP was measured through a saline-filled catheter positioned in the abdomen, and a tube was left in the left flank for intraperitoneal infusion of saline solution. An ileostomy was constructed on the right flank to allow the mucosa examination. Urinary output was monitored through a catheter inserted into the bladder. Finally, after complete hemostasis, the abdominal wall incision was hermetically closed.

### Measurements and derived calculations

Arterial, systemic, pulmonary, and central venous pressures and IAP were measured at end-expiration with zero set at the midaxillary line (Statham P23 AA, Statham, Hato Rey, Puerto Rico). Cardiac output was measured by thermodilution with 5 mL of 0°C saline solution (HP OmniCare Model 24 A 10, Hewlett Packard, Andover, Mass, USA). The mean value from three measurements taken randomly during the respiratory cycle was subsequently expressed per kilogram of body weight. Superior mesenteric and left renal blood flows were measured by an ultrasonic flowmeter (One Channel Perivascular Flowmeter, Transonics Systems Inc., Ithaca, NY, USA) and normalized to the organ weights.

Urinary output was monitored hourly and indexed to body weight. APP was calculated as MAP - IAP and renal filtration gradient (RFG) as MAP - 2 X IAP [11].

Arterial, mixed venous, and mesenteric venous PO_2_, PCO_2_, and pH were measured with an ABL5 blood gas analyzer (Radiometer, Copenhagen, Denmark), and hemoglobin and oxygen saturation with an OSM3 cooximeter calibrated for sheep blood (Radiometer). Systemic and intestinal oxygen transports and consumptions (DO_2_ and VO_2_) were calculated by standard equations and are expressed as indices of body and intestinal weight.

Intramucosal PCO_2_ was measured with a tonometer (Tonometrics Catheter, Datex-Ohmeda, Helsinki, Finland) through the use of an automated air tonometry system (Tonocap, Datex-Ohmeda, Helsinki, Finland). Its value was used to calculate intramucosal-arterial PCO_2_ (ΔPCO_2_).

Arterial lactate, [Na^+^], [K^+^], and [Cl^−^] were measured with a point-of-care analyzer (Stat Profile Critical Care Xpress, Nova Biomedical, Waltham, MA, USA). Anion gap was calculated as ([Na^+^] + [K^+^]) – ([Cl^−^] + [HCO_3_^−^]).

### Microcirculatory measurements and analysis

We evaluated the microcirculatory network in the sublingual and intestinal ileal mucosae with a MicroScan sidestream dark field (SDF) imaging device (MicroVision Medical, Amsterdam, Netherlands) [12]. For the assessment of villi, the device was gently introduced into the abdomen through the ileostomy. Careful precautions and specific steps were taken both to obtain images of adequate quality and to ensure good reproducibility. Experienced researchers performed the video acquisitions and image analyses. Steady images of at least 20 s were obtained to avoid pressure artifacts, by means of a portable computer and an ADVC110 analog-to-digital video converser (Canopus Co, San Jose, CA, USA). Clips were stored as AVI files on the hard disk to allow computerized frame-by-frame image analysis. SDF images were acquired from three different regions in the site of interest, after verifying that focus and contrast adjustment were adequate; poor-quality images were discarded. The whole sequence was used to characterize the microvascular blood flow semi-quantitatively, especially regarding the presence of stopped or intermittent flow.

The video clips were analyzed blindly and randomly. First, we used a modification of a semi-quantitative score, which distinguishes no flow (0), intermittent flow (1), sluggish flow (2), and continuous flow (3). A value was assigned to each individual vessel. The overall score, called the microvascular flow index (MFI), is the mean of the individual vessel values. For each animal, values obtained from three fields were averaged [13]. Second, we used Automated Vascular Analysis (AVA) 3.0, an image analysis software package (Academic Medical Center, University of Amsterdam, Amsterdam, Netherlands) developed for SDF video images, to determine vascular density [14]. Finally, we estimated the percentage of perfused vessels and the total and perfused vascular densities. The former was calculated from the number of vessels with flows of 2 and 3 after division by the total number of vessels and multiplication by 100. The latter was calculated from the total density, after multiplication by the fraction of perfused vessels. We also determined the heterogeneity of perfusion as the heterogeneity flow index, as the difference between the highest and lowest MFI, divided by the mean MFI [15].

In the sheep, most of sublingual vascular density (97% (SD, 1%) of total vessel length, and all villi vessels, consist of small vessels (diameter below 20 μm)) [16], so the analysis was focused on these type of vessels.

### Experimental procedure

Basal measurements were taken after a stabilization period of no less than 30 min. Then, warmed saline solution (363 ± 47 mL.kg^−1^ of body weight) was infused to reach an IAP of 20 mm Hg. IAP was continuously monitored and maintained constant. After 1 h of IAH, sheep were randomized to IAH control (*n* = 8) or IAH norepinephrine (*n* = 8) groups. In both groups, IAP was maintained for 1 additional hour at 20 mm Hg; but in the second one, MAP was increased about 20 mm Hg by means of norepinephrine to reach APP >60 mm Hg. We also included a sham group (*n* = 8) in which all surgical procedures were performed without infusing intraperitoneal saline solution. An infusion of hydroxyethyl starch 6% 130/0.4 in 0.9% NaCl (Voluven, Fresenius Kabi, Bad Homburg, Germany) was administered throughout the study to maintain the cardiac output constant and to avoid MAPs <60 mm Hg. Measurements were repeated at 60 and 120 min.

At the end of the experiment, animals were euthanized with an additional dose of pentobarbital and a potassium chloride bolus. A catheter was inserted in the superior mesenteric artery for the instillation of India ink. The dyed intestinal segments were dissected, washed, and weighed to calculate the gut indices. The left kidney was also weighed.

### Statistical analysis

Data were assessed for normality using the Shapiro-Wilk test and expressed as mean ± SD. Two-way repeated measures analysis of variance (ANOVA) was used for comparison between the three groups. Pairwise comparisons between groups were performed with unpaired *t*-test with Bonferroni correction after a significant time X group interaction. The relationship between regional blood flow and abdominal perfusion pressure was evaluated with linear regression analysis. A *P* value <0.05 was considered to be significant.

## Results

### Systemic effects

**Figure 1:**
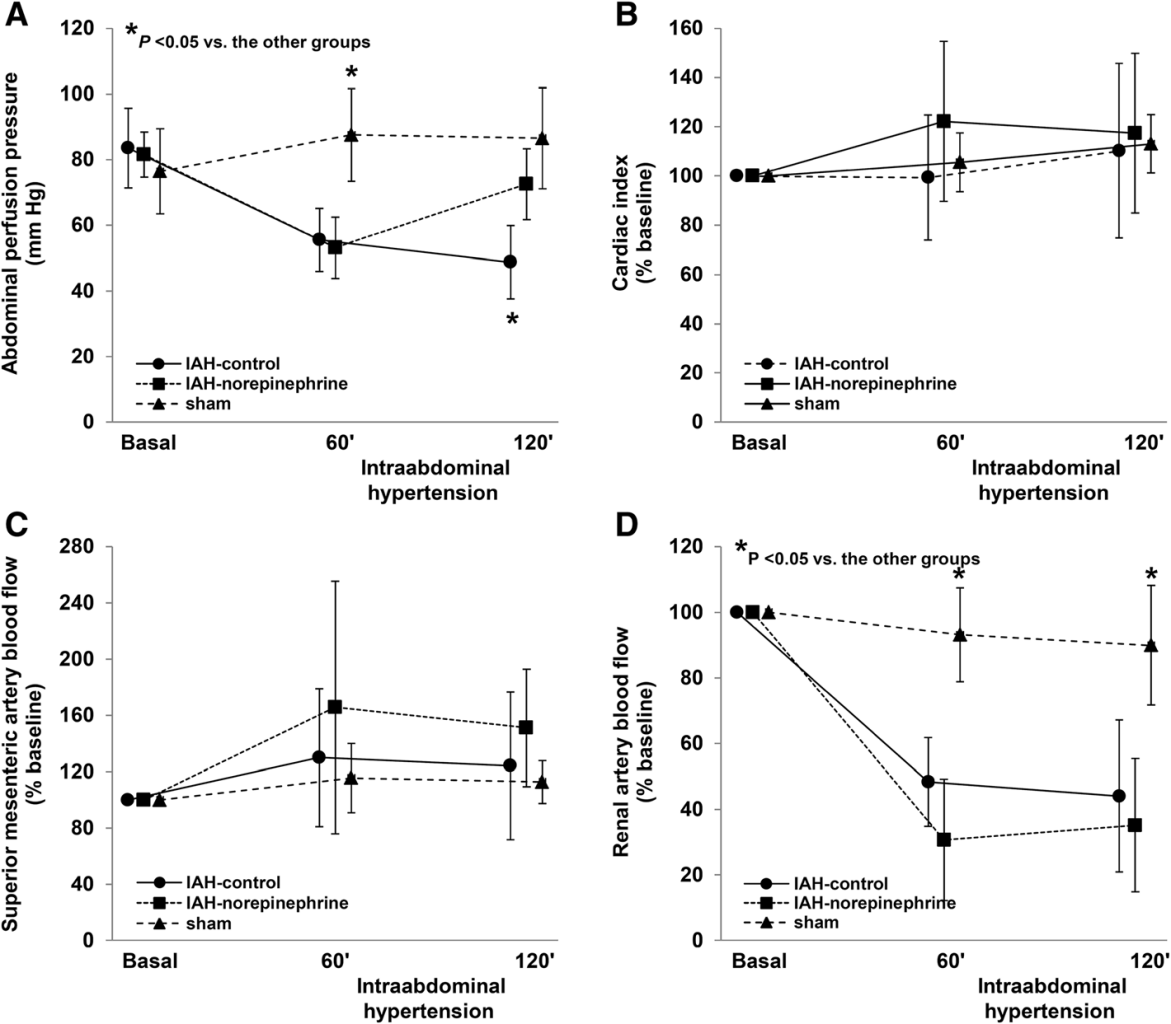
**Changes in hemodynamics variables in IAH control, IAH norepinephrine and sham groups. (A)** Abdominal perfusion pressure. **(B)** Percent of change in cardiac output. **(C)** Percent of change in superior mesenteric artery blood flow. **(D)** Percent of change in left renal artery blood flow.

There were no differences in MAP at baseline and at 60 min among groups; but at 120 min, MAP was lower in the IAH control group compared to the others. APP decreased similarly in IAH control and IAH norepinephrine groups at 60 min. At 120 min, APP in IAH norepinephrine group increased to higher values than in IAH control group, but not different from the sham group. In IAH control and IAH norepinephrine groups, pulmonary and central venous pressures were higher than in the sham group. Cardiac index was similar throughout the experiment in the three groups (Table 1 and Figure 1). The dose of norepinephrine required to reach the target MAP was 0.23 ± 0.10 μg.min^−1^.kg^−1^.

**Table 1:** **Systemic hemodynamics, oxygen transport, and acid-base variables**

			**Abdominal hypertension**	
		**Basal**	**60′**	**120′**
Mean arterial blood pressure (mm Hg)	IAH control	83 ± 12	75 ± 10	69 ± 11*
	IAH norepinephrine	81 ± 7	73 ± 9	93 ± 11
	Sham	76 ± 13	87 ± 14	87 ± 15
Heart rate (beats.min^−1^)	IAH control	177 ± 28	183 ± 51	186 ± 42
	IAH norepinephrine	173 ± 26	185 ± 45	209 ± 43
	Sham	173 ± 46	174 ± 38	184 ± 30
Cardiac index (mL.min^−1^.kg^−1^)	IAH control	122 ± 26	121 ± 41	134 ± 55
	IAH norepinephrine	96 ± 17	118 ± 42	113 ± 37
	Sham	113 ± 18	120 ± 28	127 ± 24
Mean pulmonary pressure (mm Hg)	IAH control	16 ± 4	27 ± 6	27 ± 4
	IAH norepinephrine	18 ± 7	34 ± 11	35 ± 11
	Sham	16 ± 3	18 ± 6*	18 ± 7*
Pulmonary artery occlusion pressure (mm Hg)	IAH control	3 ± 2	8 ± 4	8 ± 3
	IAH norepinephrine	4 ± 2	11 ± 5	14 ± 6*
	Sham	4 ± 2	3 ± 2*	3 ± 2*
Central venous pressure (mm Hg)	IAH control	2 ± 2	7 ± 3	6 ± 3
	IAH norepinephrine	2 ± 2	9 ± 5	11 ± 6*
	Sham	2 ± 1	1 ± 1*	2 ± 2*
Systemic vascular resistance (dynes.s.cm^−5^)	IAH control	3,071 ± 459	2,756 ± 773	2,236 ± 481
	IAH norepinephrine	3,138 ± 890	2,433 ± 1,481	2,918 ± 1,401
	Sham	2,579 ± 714	2,900 ± 984	2,674 ± 935
Pulmonary vascular resistance (dynes.s.cm^−5^)	IAH control	523 ± 195	774 ± 339	682 ± 251
	IAH norepinephrine	541 ± 247	872 ± 661	752 ± 432
	Sham	435 ± 199	525 ± 280	471 ± 267
Systemic oxygen transport (mL.min^−1^.kg^−1^)	IAH control	13.4 ± 3.1	10.2 ± 2.9	10.7 ± 4.6
	IAH norepinephrine	10.7 ± 3.0	8.4 ± 3.7	8.1 ± 3.9
	Sham	11.2 ± 3.7	10.4 ± 3.2	10.9 ± 3.1
Systemic oxygen consumption (mL.min^−1^.kg^−1^)	IAH control	6.3 ± 1.1	6.0 ± 1.2	6.4 ± 2.2
	IAH norepinephrine	5.3 ± 1.3	5.8 ± 2.3	4.7 ± 2.0
	Sham	5.0 ± 2.3	5.1 ± 2.3	5.0 ± 1.9
Arterial pH	IAH control	7.39 ± 0.07	7.27 ± 0.06	7.20 ± 0.09
	IAH norepinephrine	7.39 ± 0.04	7.22 ± 0.08	7.11 ± 0.15
	Sham	7.40 ± 0.06	7.38 ± 0.08*	7.37 ± 0.10*
Arterial PCO_2_ (mm Hg)	IAH control	35 ± 3	39 ± 5	40 ± 6
	IAH norepinephrine	35 ± 5	42 ± 3	43 ± 7
	Sham	37 ± 4	39 ± 4	38 ± 3
Arterial PO_2_ (mm Hg)	IAH control	79 ± 9	58 ± 13	58 ± 16
	IAH norepinephrine	76 ± 9	47 ± 8	55 ± 8
	Sham	75 ± 11	67 ± 18	69 ± 16
Arterial base excess (mmol.L^−1^)	IAH control	−2 ± 3	−9 ± 3	−12 ± 3
	IAH norepinephrine	−3 ± 4	−10 ± 5	−15 ± 7
	Sham	−1 ± 3	−2 ± 4*	−3 ± 5*
Arterial anion gap (mmol.L^−1^)	IAH control	12 ± 3	15 ± 5	17 ± 6
	IAH norepinephrine	11 ± 2	14 ± 4	16 ± 5
	Sham	9 ± 3	9 ± 3*	9 ± 5*
Arterial lactate (mmol.L^−1^)	IAH control	1.9 ± 0.7±	4.0 ± 2.5	6.9 ± 2.8
	IAH norepinephrine	2.2 ± 0.5	5.0 ± 2.9	6.9 ± 1.8
	Sham	1.9 ± 0.4	1.3 ± 0.6*	2.6 ± 0.6*

*P < 0.05 vs. the other groups.

In IAH control and IAH norepinephrine groups, there were comparable reductions of arterial pH and base excess, partially explained by the increase in the anion gap and in lactate concentration (Table 1). These groups required a larger volume of fluid administration to maintain cardiac output compared to the sham group (1,001 ± 246, 938 ± 170, and 376 ± 105 mL, respectively, *P* < 0.0001).

### Effects on sublingual microcirculation

None of the sublingual microcirculatory variables changed over time (Additional file 1).

### Intestinal effects

**Figure 2:**
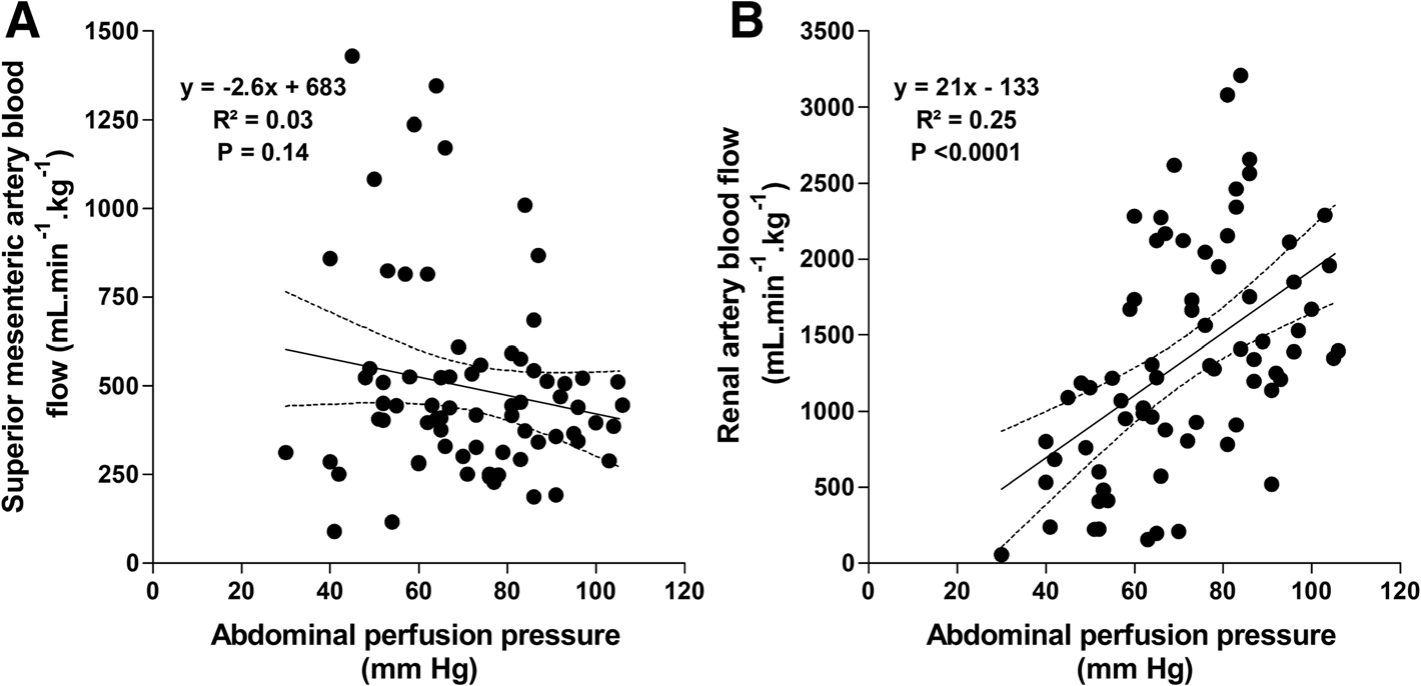
**Blood flow/perfusion pressure relationships. (A)** Correlation between superior mesenteric artery blood flow and abdominal perfusion pressure. **(B)** Correlation between left renal artery blood flow and abdominal perfusion pressure.

Superior mesenteric artery blood flow (Figure 1 and Table 2), ΔPCO_2_ (Table 2), and each villi microvascular variable remained unchanged in the three groups (Additional file 2). We found no relationship between superior mesenteric artery blood flow and APP (Figure 2).

**Table 2:** **Intestinal and renal hemodynamics, oxygen transport, and perfusion variables**

			**Abdominal hypertension**	
		**Basal**	**60′**	**120′**
Superior mesenteric artery blood flow (mL.min^−1^.kg^−1^)	IAH control	392 ± 154	524 ± 302	522 ± 362
	IAH norepinephrine	445 ± 318	633 ± 383	634 ± 401
	Sham	405 ± 115	449 ± 88	448 ± 108
Intestinal oxygen transport (mL.min^−1^.kg^−1^)	IAH control	45.2 ± 16.5	44.9 ± 24.6	43.5 ± 30.9
	IAH norepinephrine	49.4 ± 37.4	44.6 ± 32.7	45.3 ± 33.8
	Sham	38.6 ± 9.6	39.2 ± 11.4	37.8 ± 10.7
Intestinal oxygen consumption (mL.min^−1^.kg^−1^)	IAH control	16.9 ± 5.1	20.7 ± 10.8	15.8 ± 11.0
	IAH norepinephrine	17.9 ± 10.7	23.5 ± 13.4	23.7 ± 12.5
	Sham	14.8 ± 5.9	15.9 ± 7.0	15.8 ± 6.3
Intramucosal-arterial PCO_2_ (mm Hg)	IAH control	7 ± 6	9 ± 6	8 ± 5
	IAH norepinephrine	11 ± 7	12 ± 5	12 ± 8
	Sham	4 ± 4	4 ± 6	3 ± 5
Renal filtration gradient (mm Hg)	IAH control	83 ± 12	35 ± 5	29 ± 11
	IAH norepinephrine	81 ± 7	33 ± 9	53 ± 11*
	Sham	76 ± 13	87 ± 14*	87 ± 15*
Renal blood flow (mL.min^−1^.kg^−1^)	IAH control	1,906 ± 517	943 ± 416	869 ± 545
	IAH norepinephrine	1,905 ± 729	552 ± 359	620 ± 317
	Sham	1,890 ± 639	1,729 ± 510*	1,678 ± 569*
Renal oxygen transport (mL.min^−1^.kg^−1^)	IAH control	222 ± 64	81 ± 34	75 ± 43
	IAH norepinephrine	214 ± 01	38 ± 28	45 ± 29
	Sham	183 ± 58	147 ± 37*	144 ± 54*
Urinary output (mL.h^−1^.kg^−1^)	IAH control	1.2 ± 0.3	0.4 ± 0.1	0.3 ± 0.1
	IAH norepinephrine	1.1 ± 0.5	0.4 ± 0.3	0.2 ± 0.1
	Sham	1.3 ± 0.6	0.9 ± 0.5*	1.0 ± 0.6*

*P < 0.05 vs. the other groups.

### Renal effects

At 60 and 120 min of IAH, renal blood flow and urinary output were lower in IAH control and IAH norepinephrine groups, compared to the sham group (Figure 1 and Table 2). At 60 min, RFG decreased in IAH control and IAH norepinephrine groups, compared to the sham group. At 120 min, RFG increased in IAH norepinephrine group but remained lower than in the sham group. There were significant correlations between renal artery blood flow and APP (Figure 3) and between renal artery blood flow and urinary output (*R*^2^ = 0.27, *P* < 0.0001). RFG was correlated with renal blood flow (*R*^2^ = 0.39, *P* < 0.0001) and urinary output (*R*^2^ = 0.31, *P* < 0.0001).

## Discussion

The main finding of this study was the contrasting effect of IAH on renal and gut physiology. Renal blood flow and urinary output experienced a sharp decrease while intestinal flow remained unaffected, even after the increase in MAP and APP produced by the infusion of norepinephrine.

In many experimental studies about IAH, it is difficult to ascertain whether hypoperfusion of intra-abdominal organs is a direct consequence of increased IAP, or just the result of low cardiac output, a well-known deleterious effect of IAH [17,18]. IAH may decrease cardiac output by different mechanisms, but the main cause is the reduction in cardiac filling, secondary to increased intrathoracic pressure and decreased venous return from the abdomen and lower limbs [19]. In this model, cardiac output was kept constant by the infusion of fluids. In this way, we ruled out low cardiac output as the cause of intra-abdominal hypoperfusion. Although our study was not aimed at assessing the mechanisms of cardiac output reduction mediated by IAH, our sheep model showed that the increase in preload was able to preserve cardiac output. This finding highlights the relevance of avoiding hypovolemia in IAH.

Experimental research has shown controversial results about the gut involvement in IAH. Some studies describe reductions in mesenteric blood flow and mucosal microcirculation. Severe alterations in the villi microcirculation were reported with 15 mm Hg of IAP [20]. Also, rabbits exposed to nitrogen pneumoperitoneum developed reduced mucosal blood flow and increased intestinal permeability, which resulted in endotoxin release to the systemic circulation, mitochondrial damage, and necrosis of the gut mucosa [7]. Even though these studies warn about the catastrophic consequences of gut ischemia generated by IAH, it is not possible to ensure that the underlying mechanism is tissue hypoperfusion. Since cardiac output was not measured, it cannot be discarded that intestinal ischemia was the result of the compromise of systemic hemodynamics secondary to IAH, instead of increased IAP or reduced APP. Accordingly, in other studies, the changes in intestinal perfusion [21] as all as in other organs were related to the changes in cardiac output, even when MAP and APP were only slightly reduced [18]. An experimental model of intraperitoneal insufflation with CO_2_ found only subtle reductions in microcirculatory flow in the small bowel mucosa [22], which could be completely explained by decreases in cardiac output.

Accordingly, a main finding of our study, in which cardiac output was maintained stable, was the preservation of intestinal perfusion and oxygenation. We used a comprehensive approach for evaluation that included measurements of regional blood flow, oxygen transport and consumption, tissue PCO_2_, and videomicroscopy of the villi. After 2 h of an IAH of 20 mm Hg and a mean final APP lower than 50 mm Hg, all variable values were preserved. In addition, the superior mesenteric artery blood flow was not related to APP. This finding evidences the intestinal ability to autoregulate blood flow at lower driving pressures than other organs, such as the kidney. In line with our results, a previous animal study in which intestinal perfusion pressure was reduced by means of an adjustable clamp showed an ischemic threshold of only 33 mm Hg of MAP [23]. Taking everything into account, we might speculate that in IAH, intestinal oxygenation and perfusion are preserved despite the fall in APP, because the intestine has a very low limit of flow autoregulation. The maintenance of cardiac output, however, seems to be required for such preservation.

We found that IAH was associated with severe reductions of renal blood flow and urinary output in face of a preserved cardiac output, implying a decrease in the fraction of cardiac output directed to the kidney. In contrast to intestinal blood flow, renal blood flow was linearly related to APP, suggesting a limited ability for autoregulation. Experimental studies also showed that impairments in renal blood flow and function were not corrected just by increasing cardiac output, suggesting that local phenomena might be responsible [11,24]. Conversely, other animal investigations showed a direct dependence of renal blood flow on cardiac output [18,25]. These discrepancies might be related to the different models of IAH.

Distinct processes may be responsible for the decrease in glomerular filtration and oliguria in IAH: from the reduction in cardiac output, renal blood flow, and APP to direct parenchymal, cava, renal vein, or ureteral compression and neurohumoral dysfunction [3]. Although this study was not designed to explore these mechanisms, the significant correlation between urinary output and renal artery blood flow, APP, and renal filtration gradient suggests that the decreases in these variables induced by IAH were major determinants of oliguria.

In a previous study in bacteremic and non-bacteremic dogs with IAH, the restoration of APP with norepinephrine resulted in substantial but not complete reversal of the decreases in cardiac output and renal blood flow [24]. In our study, the increase in MAP induced by norepinephrine was associated with significant improvements in APP and RFG compared to IAH control group. In spite of these potentially helpful effects, renal arterial blood flow and urinary output remained as low as in the IAH control group. Although these findings might indicate that the increase in driving pressure was not able to improve perfusion, the positive correlation between APP and renal blood flow suggests that increases >20 mm Hg in MAP and APP might be needed for such purpose. In addition, the RFG was improved but not normalized by norepinephrine. The renal filtration gradient is the fluid mechanical force across the glomerulus and is equal to the difference between glomerular filtration pressure and proximal tubular pressure. The decrease in RFG seems to be crucial to explain the renal impairment during IAH [11]. Therefore, higher increases in blood pressure might have beneficial effects on renal physiology.

There is some concern about the potential detrimental effects of norepinephrine on tissue perfusion because of an excessive vasoconstriction. Indeed, septic patients with preserved sublingual microcirculation showed a detrimental response to increasing doses of norepinephrine [26]. Despite this, we noticed no harmful effects of norepinephrine on a previously well-perfused villi microcirculation.

Notwithstanding the preservation of systemic and intestinal oxygen transport and villi and sublingual microcirculation, an anion gap metabolic acidosis and hyperlactatemia ensued. The ischemia of abdominal wall muscles could play a role in the development of these abnormalities. Studies using microdialysis showed that the increase in tissue lactate-to-pyruvate ratio, a marker of anaerobic metabolism, is more pronounced in the rectus abdominis muscle than in intra-abdominal organs [27], though ischemia could have developed in territories not evaluated, such as stomach, colon, and seromuscular layer of the small intestine. In experimental IAH produced by intraperitoneal insufflation of CO_2_, small bowel mucosa was better preserved than the rest of microcirculatory gastrointestinal beds [22]. Finally, a decrease in the clearance of lactate secondary to hepatic hypoperfusion might have contributed [28].

This study has some limitations. While our model shared some characteristics with clinical conditions, IAH was not generated from capillary leakage and edema. Unfortunately, a more suitable experimental model has not yet been developed. Since only a single step in MAP and APP elevation was studied, we cannot rule out that additional increases in these variables induced by norepinephrine could further improve renal perfusion. Since larger volumes of a chloride-rich solution were infused in IAH groups, the load of chloride could play a role in the decrease of renal blood flow [29]. Also, the administration of a starch solution could have produced deleterious effects on renal function [30]**.** Other limitations are the short observation period, the lack of histology, and the small sample size. Finally, renal function was assessed only by means of urinary output. Experimental studies on IAH, however, have showed that this variable adequately tracks glomerular filtration [18,22].

## Conclusions

In this experimental model in which systemic hemodynamics was maintained by fluid resuscitation, an IAP of 20 mm Hg sustained for 2 h was associated with large decreases in renal blood flow and urinary output. In contrast, intestinal regional and microcirculatory blood flows were preserved. These findings suggest that the kidney and the gut have different autoregulatory thresholds. In addition, an increase of 20 mm Hg in MAP and APP induced by norepinephrine failed to improve kidney perfusion and function. Further studies are needed to define if larger increases in those variables might be useful.

## **Additional files**

Additional file 1:
**Changes in sublingual microcirculatory variables in IAH control, IAH norepinephrine, and sham groups.**
Additional file 2:
**Changes in intestinal villi microcirculatory variables in IAH control, IAH norepinephrine and sham groups.** **(A)** Total vascular density. **(B)** Perfused vascular density. **(C)** Proportion of perfused vessels. **(D)** Microvascular flow index. **(E)** Heterogeneity flow index.

## Abbreviations

APP: abdominal perfusion pressure
DO_2_: oxygen transport
IAH: intra-abdominal hypertension
IAP: intra-abdominal pressure
MAP: mean arterial pressure
MFI: microvascular flow index
RFG: renal filtration gradient
VO_2_: oxygen consumption
ΔPCO_2_: intramucosal-arterial PCO_2_
