# Supplementary material for: Effects of norepinephrine on tissue perfusion in a sheep model of intra-abdominal hypertension
Source: Intensive Care Med Exp. 2015 Mar 31;3:11. doi: 10.1186/s40635-015-0046-1 (PMC4513008; doi:10.1186/s40635-015-0046-1)
Supplement: Additional file 1: — Changes in sublingual microcirculatory variables in IAH control, IAH norepinephrine, and sham groups. [file 40635_2015_46_MOESM1_ESM.pdf]

**Additional file 1. Sublingual microcirculatory variables.**				
			**Abdominal hypertension**	
		**Basal**	**60′**	**120′**
**Total vascular density (mm/mm^2)**	IAH-control	27.0 ± 2.3	29.2 ± 3.1	27.8 ± 3.8
	IAH-norepinephrine	26.7 ± 2.4	28.0 ± 3.0	27.8 ± 2.5
	sham	26.8 ± 3.4	29.0 ± 3.4	28.6 ± 1.8
**Perfused vascular density (mm/mm^2)**	IAH-control	27.0 ± 2.3	29.2 ± 3.1	27.8 ± 3.8
	IAH-norepinephrine	26.6 ± 2.4	27.8 ± 2.8	27.7 ± 2.4
	sham	26.8 ± 3.4	29.0 ± 3.4	28.6 ± 1.8
**Proportion of perfused vessels**	IAH-control	1.00 ± 0.00	1.00 ± 0.00	1.00 ± 0.00
	IAH-norepinephrine	1.00 ± 0.00	0.99 ± 0.01	1.00 ± 0.01
	sham	1.00 ± 0.00	1.00 ± 0.01	1.00 ± 0.00
**Microvascular flow index**	IAH-control	3.0 ± 0.0	3.0 ± 0.0	3.0 ± 0.0
	IAH-norepinephrine	3.0 ± 0.0	3.0 ± 0.0	3.0 ± 0.0
	sham	3.0 ± 0.0	3.0 ± 0.0	3.0 ± 0.0
**Heterogeneity flow index**	IAH-control	0.01 ± 0.04	0.03 ± 0.08	0.04 ± 0.12
	IAH-norepinephrine	0.08 ± 0.20	0.07 ± 0.14	0.17 ± 0.32
	sham	0.00 ± 0.00	0.00 ± 0.00	0.00 ± 0.00
