# Supplementary material for: Effects of norepinephrine on tissue perfusion in a sheep model of intra-abdominal hypertension
Source: Intensive Care Med Exp. 2015 Mar 31;3:11. doi: 10.1186/s40635-015-0046-1 (PMC4513008; doi:10.1186/s40635-015-0046-1)

Additional file 2. Changes in intestinal villi microcirculatory variables in IAH-control, IAH-norepinephrine and sham groups. Panel A: Total vascular density. Panel B: Perfused vascular density. Panel C: Proportion of perfused vessels. Panel D: Microvascular flow index. Panel E: Heterogeneity flow index.

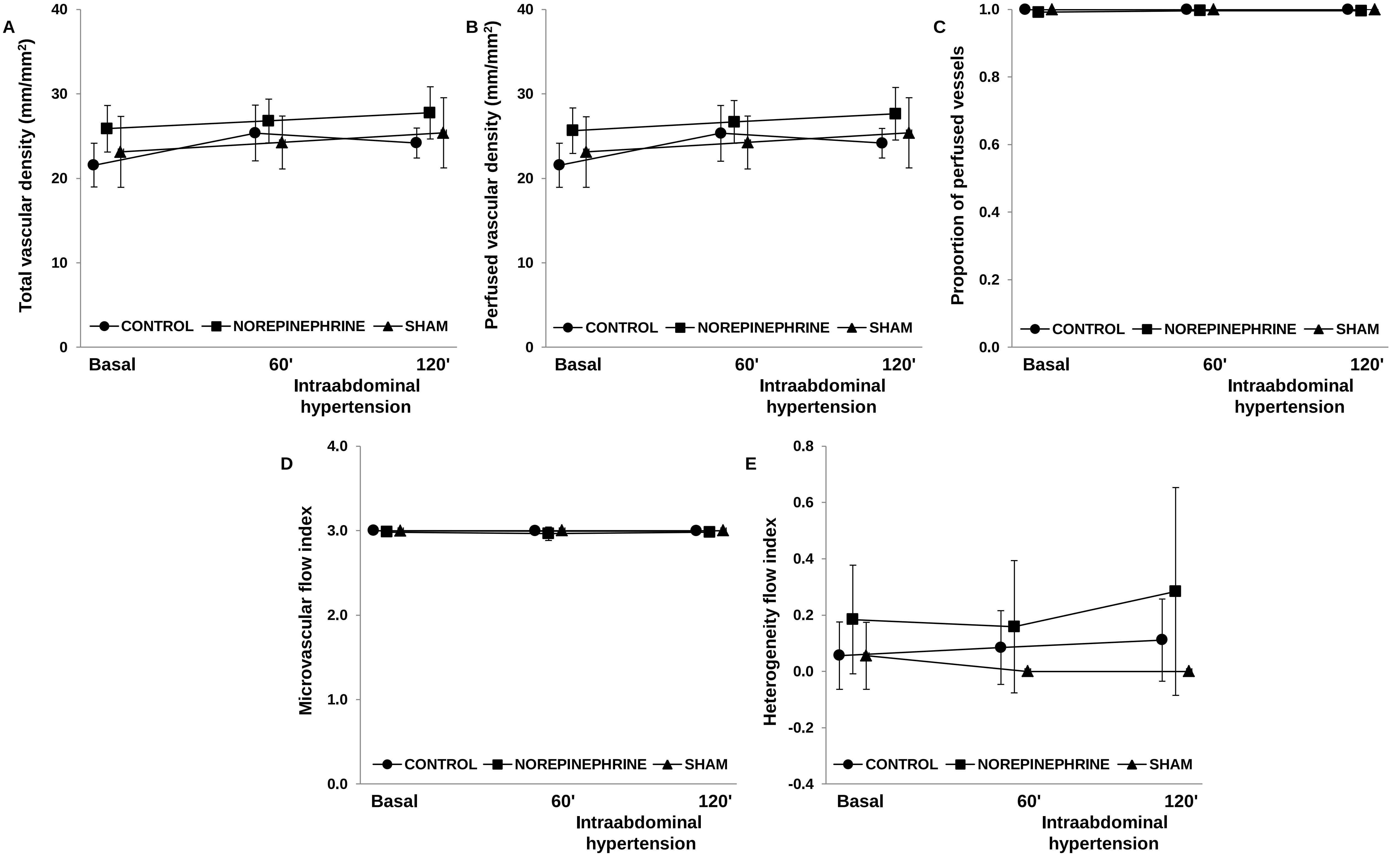

Supplement: Additional file 2: — Changes in intestinal villi microcirculatory variables in IAH control, IAH norepinephrine and sham groups. (A) Total vascular density. (B) Perfused vascular density. (C) Proportion of perfused vessels. (D) Microvascular flow index. (E) Heterogeneity flow index. [file 40635_2015_46_MOESM2_ESM.pdf]
